# Acute Cold Water-Immersion Restraint Stress Induces Intestinal Injury and Reduces the Diversity of Gut Microbiota in Mice

**DOI:** 10.3389/fcimb.2021.706849

**Published:** 2021-10-14

**Authors:** Yuan Zhang, Shuwen Wu, Yongming Liu, Jingchang Ma, Wenpeng Li, Xuexue Xu, Yuling Wang, Yanling Luo, Kun Cheng, Ran Zhuang

**Affiliations:** ^1^ Institute of Medical Research, Northwestern Polytechnical University, Xi’an, China; ^2^ Orthopedic Department of Tangdu Hospital, Fourth Military Medical University, Xi’an, China; ^3^ Department of Immunology, Fourth Military Medical University, Xi’an, China; ^4^ Library of Fourth Military Medical University, Xi’an, China

**Keywords:** gut microbiota, cold water-immersion restraint stress, intestinal injury, inflammation, mouse models

## Abstract

Growing evidence has demonstrated that stress triggers gastrointestinal (GI) disorders. This study aimed to investigate how the acute cold water-immersion restraint (CWIR) stress affects intestinal injury and gut microbiota (GM) distribution. Male C57BL/6 mice were used to establish a CWIR animal model. Hematoxylin–eosin and periodic acid–Schiff staining were performed to assess intestinal histopathological changes. Reverse transcription quantitative polymerase chain reaction (RT-qPCR) analysis and immunofluorescence staining were used to evaluate the expression of inflammatory cytokines and immune cell infiltration in the intestinal tissues. The gut permeability and intestinal occludin protein expression were determined through fluorescein isothiocyanate-dextran detection and western blot, respectively. GM profiles were analyzed *via* high-throughput sequencing of the fecal bacterial 16S rRNA genes. Results showed that CWIR induced more severe intestinal mucosal injury compared to the control, leading to a significant increase in tumor necrosis factor-α expression, but no infiltration of neutrophil and T cells. CWIR also resulted in GI disruption and increased the permeability of the intestinal mucosa. GM profiles showed that CWIR reduced GM diversity of mice compared with the control group. Specifically, aerobic and gram-negative bacteria significantly increased after CWIR, which was associated with the severity of gut injury under stress. Therefore, acute CWIR leads to severe intestinal damage with inflammation and disrupts the GM homeostasis, contributing to decreased GM diversity. Our findings provide the theoretical basis for the further treatment of intestinal disorders induced by CWIR.

## Introduction

Stress can be caused by various external or internal stimuli, leading to the emergence of strong defense systems, which may have a beneficial or harmful impact on the body (Dragos and Tanasescu, 2010; Yaribeygi et al., 2017). Acute stresses like cold-restraint, restraint, or water-immersion restraint can simulate the pathophysiology of stress-related mucosal disease (SRMD), highly prevalent in patients in intensive care units. Among these three stress models, water-immersion restraint best resembles SRMD, as previously shown by the significantly elevated ulcer index, microvascular permeability, and decreased hexosamine level (Saxena and Singh, 2017). Reportedly, serum corticosterone and glucose concentrations in rats under water-immersion restraint stress (WIRS, 23°C for 6 h) were significantly higher than those in unstressed rats (Ohta et al., 2009). Plasma epinephrine and norepinephrine levels were increased *via* the activation of the plasma enzymes in fasted rats under 6 h of WIRS (Arakawa et al., 1997). Ohta et al. demonstrated that WIRS exposure of rats induced severe oxidative stress in immune organs (Ohta et al., 2012). However, the pathogenesis of intestinal mucosal injury induced by water restraint stress remains unclear.

Mice exposed to cold-restraint stress presented gastric ulcer and inflammation (Di Cerbo et al., 2020; Higashimori et al., 2021). Further, 15-min cold exposure in mice increased their gastric phasic activity and tone, whereas mice exposed to chronic stress presented with no gastric response (Gourcerol et al., 2011). A recent study also revealed that cold stress can induce neuronal autophagy mediated by corticosterone excess in the hippocampus in C57BL/6 mice (Xu et al., 2019). Moreover, increased plasma corticosterone levels following exposure to cold water stress may be modulated by MAPK and mTOR signaling pathways (Feng et al., 2021). Cold water-immersion restraint (CWIR) stress was found to be associated with gastric mucosal lesions (Shian et al., 1995), but the evidence for its related intestinal injury is scarce. Considering that gut microbiota (GM) is an essential factor influencing intestinal health (Wan et al., 2019), the present study mainly investigated possible mechanisms and microbiome alterations underlying acute CWIR stress-induced intestinal mucosal damage. We found that acute CWIR stress led to severe intestinal mucosal injury with increased intestinal permeability. The GM diversity was significantly reduced after CWIR administration, leading to imbalanced aerobic to anaerobic proportion.

## Materials and Methods

### Animals

We obtained 8–10 week old male C57BL/6 mice (weighing 24–25 g) from the Animal Center of the Fourth Military Medical University. Mice were housed in a specific pathogen-free level laboratory under standard conditions (22°C–23°C, 12 h light/dark cycle, and 60% ± 10% humidity) and provided with water and food. In order to facilitate the microbiota study, the mice were rotated within different cages (5 mice per cage) for more than two weeks after weaning at the fourth week after birth. Then the mice were randomly divided into control and CWIR groups for further research. The inter-cage rotation and randomized grouping were carried out to mitigate the potential cage effects on the intestinal microbiota of mice (Goodrich et al., 2014; Caruso et al., 2019; Yang et al., 2020). All experimental protocols and animal handling procedures were conducted following the Care and Use of Laboratory Animals Guide. This study was approved by the Ethical Committee on Animal Experimentation of Northwestern Polytechnical University and Fourth Military Medical University.

### Acute CWIR Stress Model

Mice were fasted for overnight and restrained in polyvinyl chloride tubes, as a protocol previously reported with slight modifications (Shian et al., 1995; Szuran et al., 2000; Popova et al., 2006). The tube with the animal was vertically immersed to the level of the sternum xiphoid in water maintained at 10°C ± 1°C for 1 h. Mice subjected to the unstressed condition underwent the same procedure without restraint and cold-water exposure (Landeira-Fernandez, 2015; Saxena and Singh, 2017). After acute CWIR, mice were immediately sacrificed, and their serum and intestine tissues were obtained for further examination.

### Intestinal Morphology

The morphometric analysis of the small intestine was performed as previously described (Lin et al., 2020). Mice were sacrificed, and the small intestinal tissues were carefully dissected and immersed in 4% paraformaldehyde. After being embedded in paraffin, tissues were cut into sections (5 µm). Intestinal tissues were subjected to hematoxylin–eosin (HE) and periodic acid–Schiff (PAS) staining. For the HE staining, five representative villi or crypt per slide were randomly selected and measured. Villus height, crypt depth, and the V/C ratio were measured from each villus set and crypt in the duodenum, jejunum, and ileum. For PAS staining analysis, the average number of goblet cells per entire enterocyte was calculated in each group.

### Gut Leakage Determination

Intestinal permeability defect was determined through serum detection of fluorescein isothiocyanate dextran (FITC-dextran FD4; Sigma-Aldrich, St. Louis, MO, USA), a nonabsorbable high-molecular-weight molecule, after the oral administration of 12.5 mg FITC-dextran at 2 h after CWIR, as previously reported (Guo et al., 2012; Sae-Khow et al., 2020). A fluorospectrometer (microplate reader; Thermo Fisher Scientific, Waltham, MA, USA) was used to measure the serum FITC-dextran based on a standard curve of a reference.

### Western Blot

Intestinal tissues were rapidly homogenized in liquid nitrogen and stored at −80°C prior to western blot analyses. Total protein was extracted using a RIPA lysis buffer, and the total protein concentration was determined using a BCA kit, according to the manufacturer’s instructions (Beyotime, Shanghai, China). Proteins were separated using SDS-PAGE and transferred onto PVDF membranes (Millipore, Bedford, MA, USA). Membranes were blocked in 5% bovine serum albumin for 1 h before incubation with occludin (sc-133256, Santa Cruz Biotechnology, Santa Cruz, CA, USA), claudin-1 (AF6504, Beyotime Biotechnology Co., Shanghai, China), and β-actin primary antibodies (AF5001, Beyotime Biotechnology). After being washed with TBS-T, the membranes were incubated with HRP-conjugated secondary antibodies (G-21040, Thermo Fisher Scientific) for 2 h at 4°C. Protein signals were detected using a StarSignal system (GenStar, Beijing, China). Results were obtained from four separate experiments.

### Reverse Transcription Quantitative Polymerase Chain Reaction(RT-qPCR)

Total RNA was extracted from intestinal tissues using TRIGene reagent (GenStar), and converted to cDNA using the Hifair II 1st Strand cDNA Synthesis Kit (Yeasen Biotechnology, Shanghai, China). qPCR was performed using SYBR qPCR Master Mix (GenStar). Relative mRNA levels were calculated *via* normalization to β-actin’s level. Relative gene expression was analyzed based on the fold change (the 2−ΔΔCt method).

### Immunofluorescence Staining

Immunofluorescence staining of tissues was performed on paraffin sections as previously described (Mescher et al., 2017). The tissue sections were incubated with primary antibodies (Ly6G: GB11229, Servicebio, Wuhan, China; CD4: bs-0766R, bioss Inc., Beijing, China; TNF-α: 346654, Zen bioscience, Chengdu, China; and IL-1β: 516288, Zen bioscience) overnight at 4°C in a humidified chamber. Following this, incubation with Cy3–conjugated secondary antibodies and DAPI (Thermo Fisher Scientific) were performed for 1 h at room temperature in an antibody buffer before washing and mounting in an anti-quenching medium (Thermo Fisher Scientific). Images were captured using a fluorescent microscope (EVOS M7000 Cell Imaging Systems, Thermo Fisher Scientific).

### DNA Extraction and 16S Ribosomal RNA Sequencing

Fecal samples were collected by using standardized collection procedures. Four samples per group were used for 16S rRNA sequencing. Microbial DNA was extracted and quantified. After, the V3–V4 hypervariable region was amplified using 341F (5’-CCTACGGGNGGCWGCAG-3’) and 806R (5’-GGACTACHVGGGTATCTAAT-3’) primers. Sequencing was performed on a Illumina PE250 system by Gene Denovo Biotechnology Co., Ltd (Guangzhou, China). The raw data were quality-filtered using Usearch or DADA2 software. Bioinformatic analysis was performed using Omicsmart, a real-time interactive online platform for data analysis (www.omicsmart.com). Alpha diversity on Operational Taxonomic Units (OTU) levels was measured by Sob, Chao1, Shannon, and Simpson indexes and compared using Welch’s t-test within CWIR or control groups. Beta diversity was assessed using unweighted unifrac distance. Principal coordinates analysis (PCoA) was conducted according to the distance matrices. The link between groups of mice and bacterial microbial profiles was assessed using an Adonis test. The functional composition and phenotype of the intestinal metagenome were predicted using Phylogenetic Investigation of Communities by Reconstruction of Unobserved States (PICRUSt2) and BugBase.

### Statistical Analysis

Statistical data were analyzed using GraphPad Prism 6.0 (GraphPad Software, La Jolla, CA, United States). Data are presented as the mean ± standard error of mean (SEM). Welch or Student’s t-test was used to compare values between the two groups. Statistical significance was accepted at *P* < 0.05.

## Results

### CWIR Induces Intestinal Mucosa Injury Histologically

**Figure 1:**
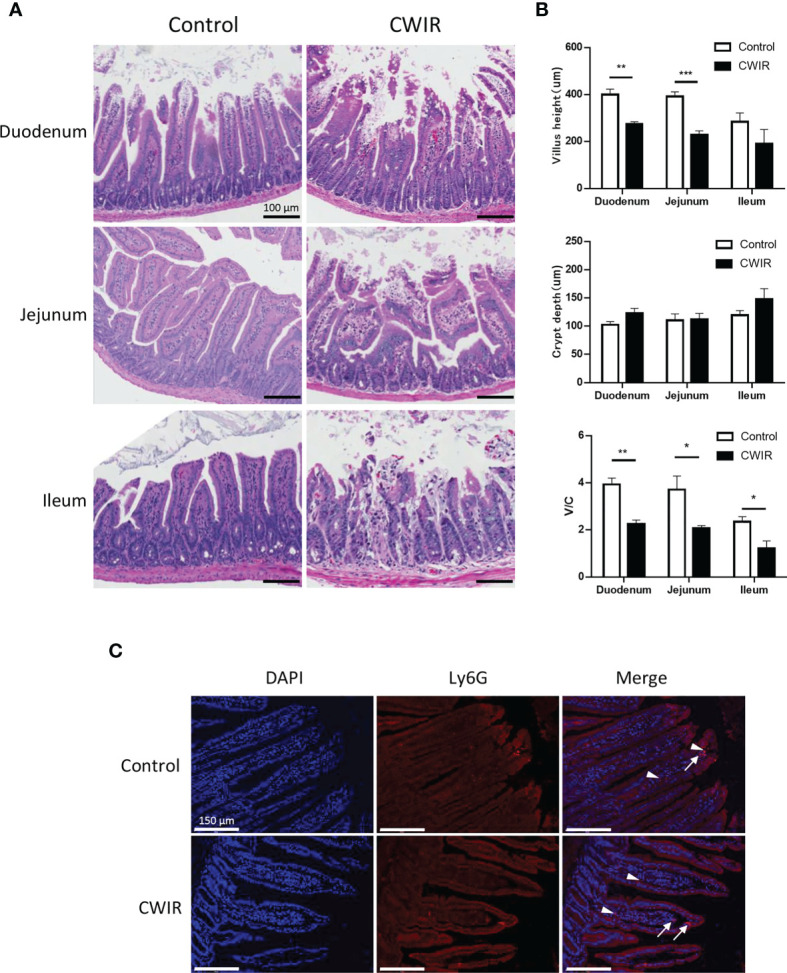
Cold water-immersion restraint (CWIR) induces gut mucosal barrier disruption in the small intestine in mice. Different segments of the small intestine were harvested 1 h post CWIR. **(A)** Hematoxylin–eosin (HE) staining of mouse intestinal tissues (magnification, 200×). Microscopic examination of the small intestinal mucosa revealed marked villous thinning, shrinkage, and disorganization in CWIR mice. Scale bar = 100 μm. **(B)** Villus height, crypt depth, and the villus/crypt (V/C) ratio were measured in the duodenum, jejunum, and ileum in mice after CWIR administration or control. All data are shown as mean ± SEM (n = 4–5 per group). *P < 0.05, **P < 0.01, ***P < 0.001 *vs.* WT control. **(C)** Representative anti-Ly6G immunofluorescent photomicrographs of the duodenum. White arrow indicates intestinal neutrophil infiltration (Ly6G positive, red, n = 3 per group). White arrowhead depicts erythrocyte-nonspecific staining, without DAPI nuclear staining. Scale bar = 150 μm.

**Figure 2:**
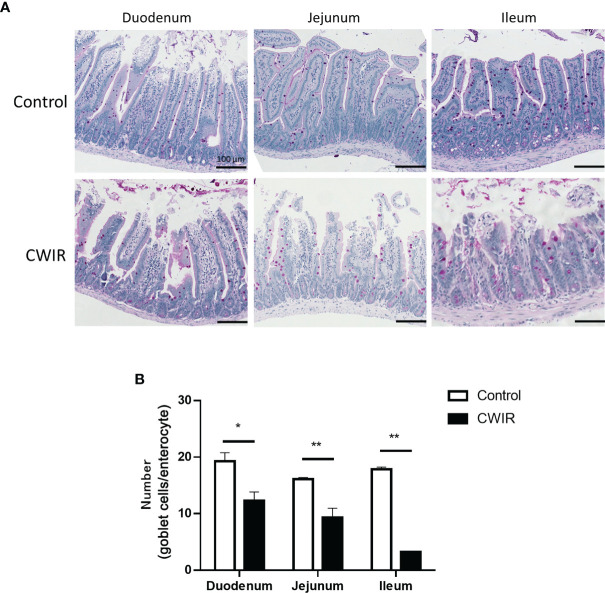
Histology of mouse intestine stained with periodic acid-Schiff (PAS) reagent. **(A)** Representative images of PAS-stained goblet cells in the small intestine (magnification, 200×). PAS stains carbohydrates in pink color. Scale bar = 100 μm. **(B)** Numbers of goblet cells analyzed on the PAS-stained sections of the duodenum, jejunum, and ileum of mice following CWIR or control treatment. All data are shown as the mean ± SEM (n = 4–5 per group). *P < 0.05, **P < 0.01 *vs.* WT control.

As presented in **Figure 1A**, histological changes in intestinal tissue sections were detected by HE staining. The intestinal mucosa in the control group exhibited a complete structure, which was disrupted by CWIR treatment (**Figure 1A**). In comparison with the control mice, CWIR mice showed significantly decreased villus heights in the duodenum and jejunum tissues, and notably reduced V/C ratios in the duodenum, jejunum, and ileum (**Figure 1B**). However, the crypt depth in the intestinal tissue sections was not different between the two groups (**Figure 1B**). We further performed immunofluorescence staining of the duodenum with anti-Ly6G and CD4 antibodies to detect neutrophil and T cell infiltration. As shown in **Figure 1C** and **Supplementary Figure 1A**, there was no difference in neutrophil and T cell infiltration between WT and CWIR mice. PAS staining showed that the goblet cells (red) were dispersed among the epithelial cells, mainly in the lower half of the villi (**Figure 2A**). Compared to the controls, mice subjected to CWIR exhibited significantly reduced goblet cell numbers in all the intestinal segments (**Figure 2B**).

### CWIR Increases Intestinal Inflammation and Intestinal Mucosal Permeability

**Figure 3:**
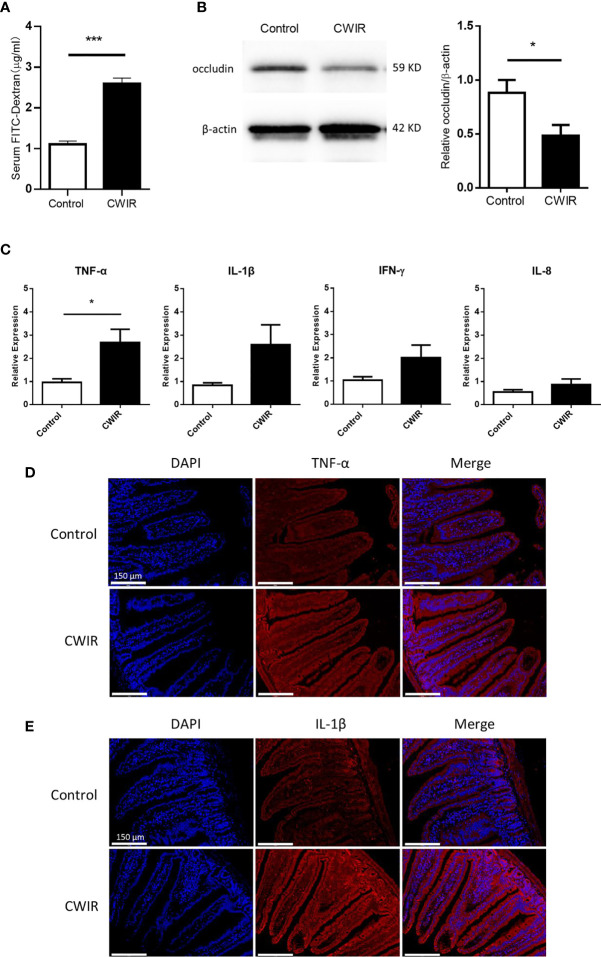
Effect of CWIR on the intestinal barrier function. **(A)** gastrointestinal (GI) permeability barrier defect, as determined by fluorescein isothiocyanate (FITC)-labeled dextran detection in CWIR injury mice or control mice. Leakage of FITC-dextran from the gut was measured (in serum) 2 h post oral administration. n = 10-11 mice per group, ***P < 0.001 *vs*. WT control, Student’s *t*-test was used to compare the values between the two groups. **(B)** Representative western blot image and protein quantification to detect occludin, a tight junction molecule, in the small intestine of CWIR injury mice or control mice. n = 5 per group, *P < 0.05 *vs*. WT control. **(C)** mRNA levels of proinflammatory cytokines in the small intestine. All data are shown as the mean ± SEM, Student’s *t*-test was used to compare the values between the two groups. *P < 0.05 *vs*. WT control, n = 4–6 per group. **(D)** Representative anti-TNF-α immunofluorescent photomicrographs of the duodenum. TNF-α-specific antibodies were detected using Cy3-conjugated secondary antibodies (red), n = 3 per group; Scale bar = 150 μm. **(E)** Representative photomicrographs of immunofluorescence against IL-1β of the duodenum. IL-1β-specific antibodies were detected using Cy3-conjugated secondary antibodies (red), n = 3 per group; Scale bar = 150 μm. Nuclei were counterstained with DAPI.

To assess whether CWIR contributed to gastrointestinal permeability defects, gut leakage was measured through FITC-dextran detection in the serum. As shown in **Figure 3A**, the FITC-dextran level in the plasma of the CWIR group was remarkably higher than that in the control group (*P* < 0.001). Western blot analysis also showed increased damage of the integrity and tight junctions of the intestine in CWIR mice, as demonstrated by the significant reduction of occludin protein expression (**Figure 3B**). However, the control and CWIR groups did not show any differences in terms of claudin-1 expression in the intestine (**Supplementary Figure 1B**). Besides, RT-qPCR analysis indicated that acute CWIR significantly increased the level of inflammatory factor tumor necrosis factor-alpha (TNF-α) in small intestine tissues. Although interleukin (IL)-1β expression was increased, the increase was not statistically significant. However, IL-8 and interferon (IFN)-γ levels did not change significantly after CWIR (**Figure 3C**). These findings were also confirmed by further immunofluorescence staining analysis for TNF-α and IL-1β in the small intestinal tissues (**Figures 3D, E**).

### CWIR Decreases GM Diversity

**Figure 4:**
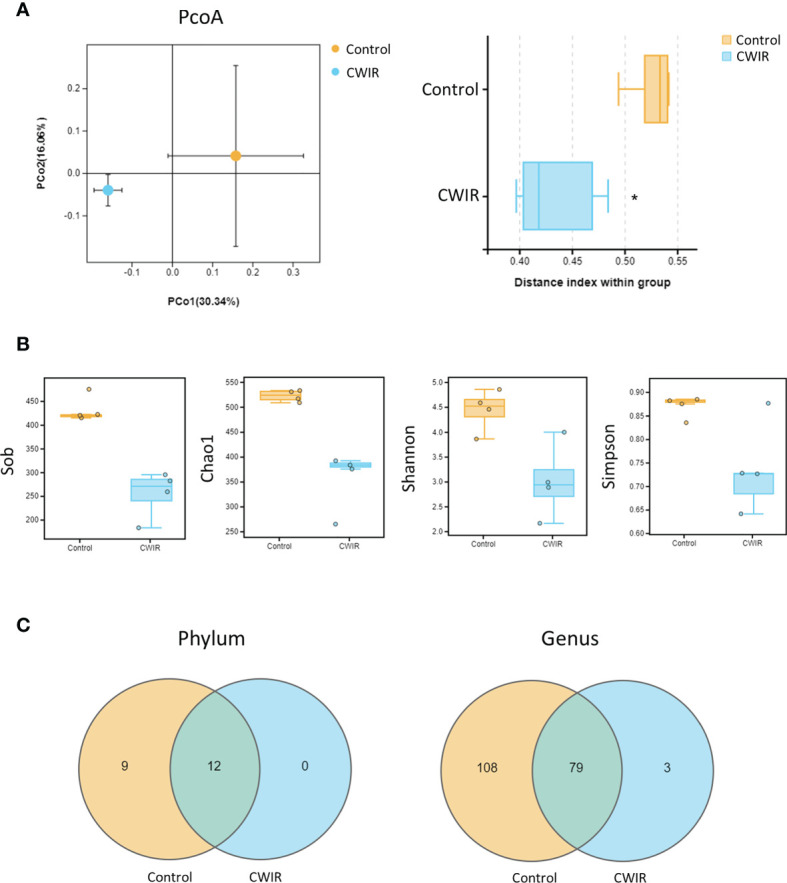
Effect of CWIR on the gut microbiota in mice. **(A)** Principal coordinate plot (PCoA) of the unweighted unifrac distance at the Operational Taxonomic Units (OTU) level. Significance between groups was analyzed by Welch’s t-test. The PCoA plot revealed an obvious separation between the two groups (Adonis: P = 0.021). **(B)** The alpha diversity indexes revealed significantly decreased ecological diversity in the fecal microbiome in CWIR mice compared with control mice. **(C)** Effects of CWIR on the number of intestinal mucosa bacterial OTUs. The Venn diagram represents the number of shared and unique OTUs for the two groups at the phylum and genus levels (through Omicsmart online platform). n = 4 per group. *P < 0.05 *vs*. Control.

To characterize GM variations in mice intestine after CWIR, 16S rRNA amplicon libraries were constructed and sequenced from cecal content, for each group. The analysis of beta diversity and alpha diversity indicated a clear variety in the intestinal flora. The PCoA plot revealed an obvious separation between the two groups, confirming that the composition of the fecal microbiota differed after CWIR stress (Adonis: P = 0.021) (**Figure 4A**). Alpha diversity was measured through Sob, Chao1, Shannon, and Simpson indices. Sob and Chao1 indicate the richness of species, whereas Shannon and Simpson emphasize the richness and evenness in a region. It was shown that CWIR treatment significantly reduced the overall bacterial richness in the gut (**Figure 4B**). Except for 12 shared phyla and 79 shared genera, there was no unique phyla and only three unique genera were observed in the CWIR samples, compared to the control (**Figure 4C**). These results suggested that acute CWIR triggered GM dysbiosis, leading to a decreased microbiota diversity.

### CWIR Affects the GM Abundance at Phylum and Genus Levels

**Figure 5:**
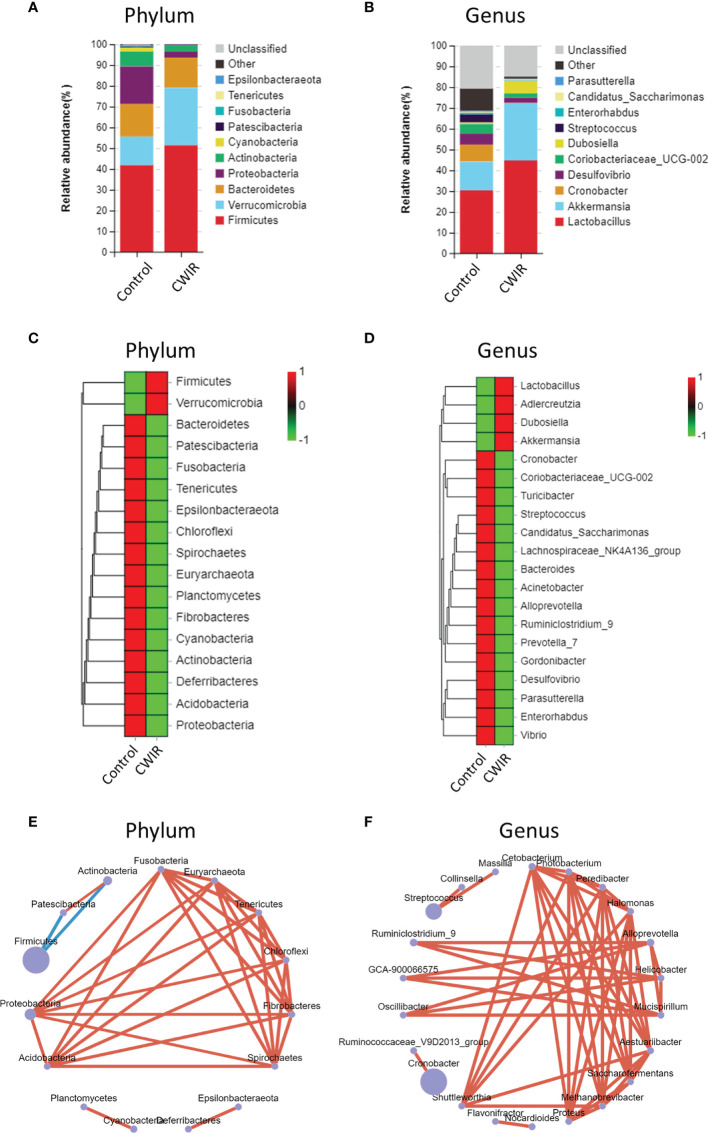
Differences in relative abundance of bacterial flora. **(A, B)** Barplot analysis of the relative abundance in the fecal microbiota of control and CWIR mice at the phylum and genus level by average. Top 10 species in the mean abundance of all samples were shown in detail, other known species were classified as others, and unknown species were marked as unclassified. **(C, D)** Relative abundances of intestinal bacteria were clustered as a heatmap at the phylum and genus level. **(E, F)** Co-occurrence bacterial networks were established from 16S sequences from CWIR fecal samples based on the abundance table. Pearson correlation coefficient was calculated, and the network diagram was displayed at the phylum level and genus level. The size of each node is proportional to the relative abundance. The lines in red and blue denote positive and negative correlations, respectively. n = 4 per group.

The enrichment of distinct bacterial communities in each group at phylum and genus levels was assessed. As shown in **Figure 5A**, Firmicutes, Proteobacteria, and Bacteroidetes were the most abundant in the normal control group, as previously reported (Long et al., 2020), while CWIR remarkably increased Verrucomicrobia and decreased Proteobacteria abundance. At the genus levels, Lactobacillus (30.41%) was the dominant species in the normal group, followed by Akkermansia (13.79%), and Cronobacter (8.21%). CWIR increased the relative abundance ratio of beneficial bacteria Lactobacillaceae and Akkermansia (belongs to the phylum Verrucomicrobia) (**Figure 5B**). Relative abundance alterations at phylum and genus levels in two groups were also clustered, as shown in **Figures 5C, D**, respectively. These data indicated that CWIR-induced intestinal injury may change the composition of intestinal flora, rather than reducing beneficial bacteria. Bacterial co-occurrence networks in the CWIR samples showed that phylum Proteobacteria and Acidobacteria, and genus Shuttleworthia were the essential taxa exerting functions in the intestine (**Figures 5E, F**).

### CWIR Alters the Microbiome Phenotypes in Mice Intestine

**Figure 6:**
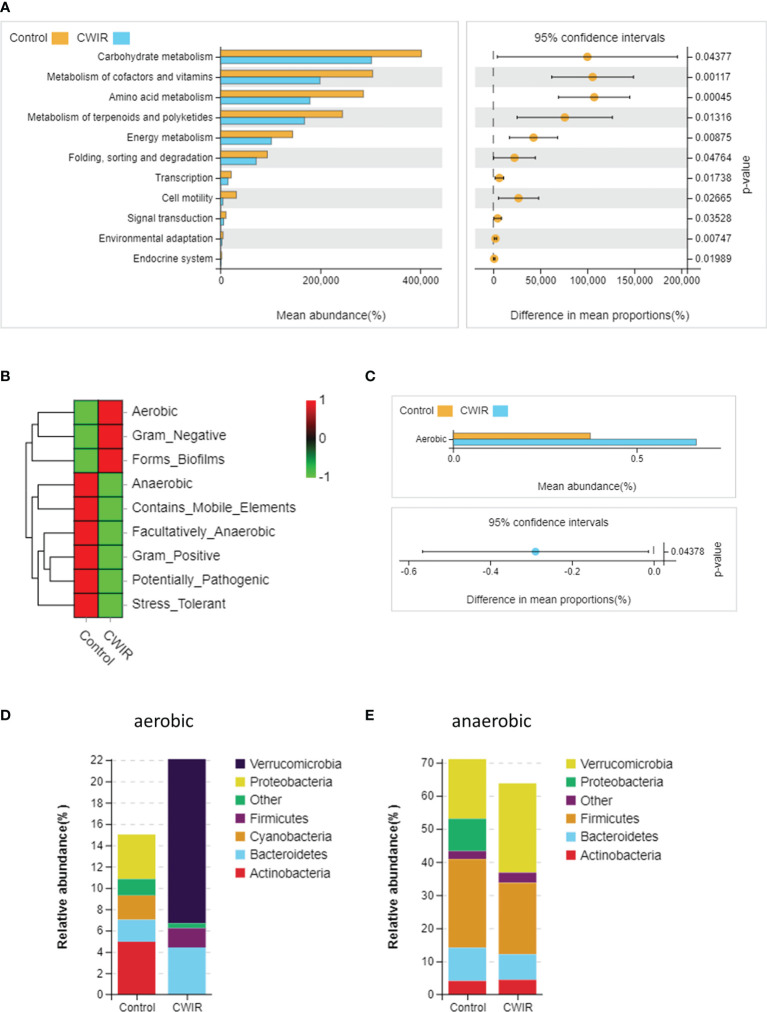
The functional capacities were predicted based on 16S data using PICRUSt2 and BugBase. **(A)** PICRUSt2 was used to predict the function of gut microbiota at level-2. **(B)** Heatmap of microbial community phenotypes and the corresponding bacterial contributions (BugBase). **(C)** Statistical significance of aerobic microbiota in the two groups was determined by Welch’s t-test. **(D, E)** Relative abundance plots of aerobic and anaerobic bacteria in the control and CWIR samples at the phyla level (BugBase). n = 4 per group.

Furthermore, Phylogenetic Investigation of Communities by Reconstruction of Unobserved States 2 (PICRUSt2) was used to predict the Encyclopedia of Genes and Genomes (KEGG) pathways associated with components of the GM. A majority of functional biomarkers was enriched in metabolic pathways. Compared to the control, CWIR reduced the metabolism in the following KEGG groups: “carbohydrate metabolism”, “metabolism of cofactors and vitamins”, “amino acid metabolism”, “metabolism of terpenoids and polyketides”, and “energy metabolism” (**Figure 6A**). Besides, 16S OTUs in the two groups were analyzed by BugBase to predict their microbiome phenotypes. In comparison with the control, CWIR samples contained increased microbiota related to aerobic (P = 0.044), gram-negative, and biofilm-forming, with reduced anaerobic, gram-positive, and stress-tolerant microbial communities (**Figures 6B, C**). Moreover, we assessed the bacterial abundance annotated in aerobic and anaerobic phenotypes, at a phyla level in each group. For aerobic bacteria, CWIR treatment decreased relative bacterial abundance in the Proteobacteria, Cyanobacteria, and Actinobacteria, but increased Bacteroidetes, Firmicutes, and Verrucomicrobia (**Figure 6D**). For anaerobic bacteria, relative abundance in the Bacteroidetes, Firmicutes, and Proteobacteria were reduced, but Verrucomicrobia increased a lot (**Figure 6E**). These results suggested that the CWIR administration disrupted the balance between aerobic and anaerobic bacteria in the mice intestine.

## Discussion

Restraint stress was initially developed by Bonfils (1993) and since then it has been widely used to mimic psychological stress (Bonfils, 1993; Bali and Jaggi, 2015). The role of stress in the induction of cardiovascular diseases, brain injury, skin and GI diseases have been previously documented (Caso et al., 2008; Kumar et al., 2020; Ren et al., 2021). Schultz et al. demonstrated that restraint stress damages mice intestine, leading to aggravated inflammatory disorders of colitis (Schultz et al., 2015). Additionally, the activation of stress-induced HPA axis and sympathetic nervous system is known to cause dysregulation of GI hormones and increase oxidative stress (Bali and Jaggi, 2015). Stress can induce the release of norepinephrine in the hypothalamic paraventricular nucleus, pituitary-adrenocortical, contributing to increased sympathoadrenal activity (Pacak et al., 1995). Also, it is known to cause dysregulation of GI hormones, resulting in excessive production of free radicals and increased oxidative stress (Shian et al., 1995; McIntosh and Sapolsky, 1996; Bali and Jaggi, 2015). These effects are part of the regulation on the physiological functions of the intestine induced by stress. In this present study, acute CWIR stress caused severe histological damage in the intestinal mucosa, combined with increased expression levels of inflammatory indicators, especially TNF-α. However, the infiltration of neutrophil and T cell had no big difference after CWIR stress. The intestinal mucosa forms the most extensive contact surface between the body and the intestinal cavity, and functions as a barrier to maintain body health, through rapidly renewal and prevention of possible invasion of harmful substances and pathogens (Camilleri et al., 2012; Sanchez de Medina et al., 2014). In addition, CWIR stress induced gastrointestinal disruption and increased intestinal mucosal permeability.

The GM is a diverse and dynamic microbes’ population with extensive and essential interactions with the digestive, immune, and nervous systems (Huo et al., 2017; Marazziti et al., 2021). The complex bidirectional communication systems between the GI tract and the brain were initially termed “gut-brain axis” and then renamed “microbiota-gut-brain axis”, considering the pivotal role of GM in sustaining local and systemic homeostasis (Lavelle and Sokol, 2020; Banfi et al., 2021). For example, irritable bowel syndrome (IBS), a functional GI disease, is related to the changes of microbiota–gut–brain axis, usually manifested in stress or enteric infection. IBS is characteristic of increased intestinal permeability, disrupted GM, and inflammation. Here we found that CWIR also altered the GM, causing significantly decreased microbiota richness. After comparing the differences of candidate taxa in both groups at phylum and genus levels, it was revealed that CWIR markedly reduced fecal bacterial species compared to the control.

Moreover, PICRUSt2 analysis predicted that CWIR significantly downregulated metabolic pathways such as “carbohydrate metabolism”, “metabolism of cofactors and vitamins”, and “energy metabolism”. Vitamins have been demonstrated to have well-established roles in bacterial metabolism and can influence the composition of microbiota community (Ellis et al., 2021). A previous study indicated that WIRS led to increased concentration of gastric mucosal lipid peroxide and myeloperoxidase activity, but decreased the gastric vitamin E (Ohta et al., 2005). These findings suggested that CWIR may lessen vitamin concentrations, thereby reducing their related metabolism. In addition, intestine is an anaerobic environment, and the loss of anaerobic bacteria or overgrowth of aerobic pathobiont would directly contribute to the disruption of normal intestinal function. Fecal aerobic bacteria are composed of gram-positive bacteria—such as *Staphylococcus aureus* and *Staphylococcus epidermidis*—and gram-negative bacteria—such as *Escherichia coli* and *Pseudomonas aeruginosa*. Our results indicated that gram-positive bacteria were decreased and gram-negative bacteria elevated in the CWIR samples. Since gram-negative bacteria in gut are an endogenous source of endotoxins, these alterations may also be associated with the severity of gut inflammation. Also, microbiota related to stress tolerance were depleted in CWIR feces, consistently with the performance after stress. The low levels of anaerobic bacteria after CWIR also contributed to significantly enhanced aerobic-related phenotype of microbiome in mice intestine. In summary, acute stress altered intestinal function as well as GM homeostasis in mice.

Acute CWIR stress caused severe intestinal injury with increased intestinal inflammation and barrier disruption. As GM presents a plastic response to various kinds of physicochemical stress, we found that fecal bacteria were rapidly remodeled in this acute CWIR stress mouse model, as confirmed by the significantly reduced microbiota diversity. These findings provide a theoretical basis for the further treatment of CWIR-related intestinal disorders.

## Data Availability Statement

The raw data supporting the conclusions of this article will be made available by the authors, without undue reservation.

## Ethics Statement

The animal study was reviewed and approved by Ethical Committee on Animal Experimentation of Northwestern Polytechnical University and Fourth Military Medical University.

## Author Contributions

YZ: manuscript drafting, study design, and statistical analysis. SW, YML, JM, and WL: completed all laboratory work. XX and YW: helped plan and coordinate the study. KC: mice genotyping. YLL: manuscript drafting preparation. RZ study design and administration support. All authors contributed to the article and approved the submitted version.

## Funding

This investigation was supported by the Medical Science and Technique Foundation for Fostering Young Scientist (18QNP026), Funding Program for Medical Science Development of FMMU (2018HKPY02), and Natural Science Basic Research Program of Shaanxi (Program No. 2021JM-081).

## Conflict of Interest

The authors declare that the research was conducted in the absence of any commercial or financial relationships that could be construed as a potential conflict of interest.

## Publisher’s Note

All claims expressed in this article are solely those of the authors and do not necessarily represent those of their affiliated organizations, or those of the publisher, the editors and the reviewers. Any product that may be evaluated in this article, or claim that may be made by its manufacturer, is not guaranteed or endorsed by the publisher.

## Supplementary Material

Supplementary Figure 1
**(A)** Representative anti-CD4 immunohistochemistry photomicrographs of duodenum. CD4-specific antibodies were detected with Cy3-conjugated secondary antibodies (red), Nuclei were counterstained with DAPI. 1 h CWIR treatment may not a sufficient time to observe significant T cell infiltration, only erythrocytes showed unspecific staining (white arrowhead). Scale bar = 150 μm, n = 3 per group. **(B)** Representative western blot image and protein quantification to detect tight junction protein claudin-1 in small intestine of CWIR injury mice or control mice. n = 6 per group.
