# Supplementary figures and images for: Acute Cold Water-Immersion Restraint Stress Induces Intestinal Injury and Reduces the Diversity of Gut Microbiota in Mice
Source: Front Cell Infect Microbiol. 2021 Oct 14;11:706849. doi: 10.3389/fcimb.2021.706849 (PMC8551804; doi:10.3389/fcimb.2021.706849)

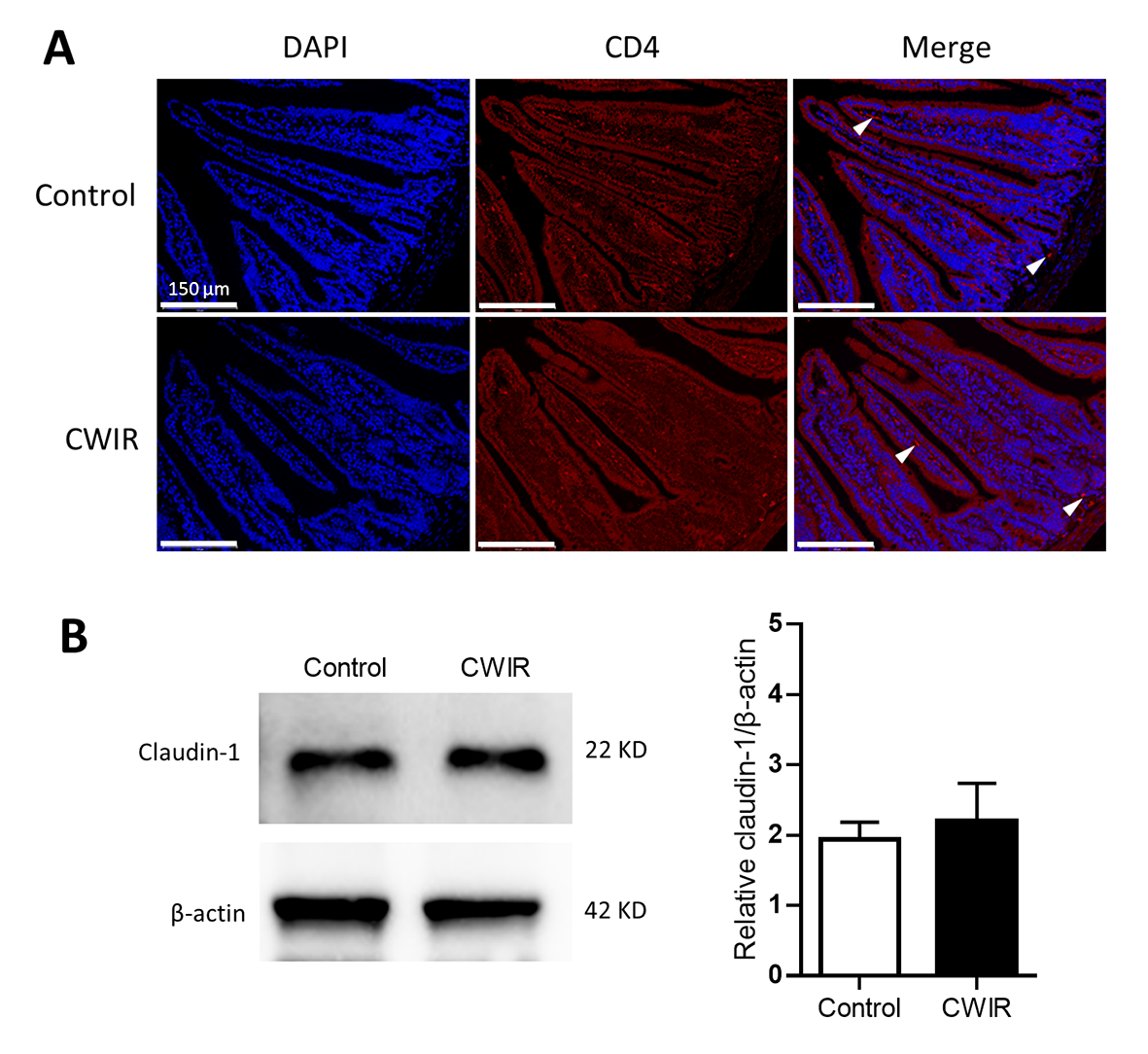

Supplement: Supplementary Figure 1 — (A) Representative anti-CD4 immunohistochemistry photomicrographs of duodenum. CD4-specific antibodies were detected with Cy3-conjugated secondary antibodies (red), Nuclei were counterstained with DAPI. 1 h CWIR treatment may not a sufficient time to observe significant T cell infiltration, only erythrocytes showed unspecific staining (white arrowhead). Scale bar = 150 μm, n = 3 per group. (B) Representative western blot image and protein quantification to detect tight junction protein claudin-1 in small intestine of CWIR injury mice or control mice. n = 6 per group. [file Image_1.tif]
